# Yoga for Essential Hypertension: A Systematic Review

**DOI:** 10.1371/journal.pone.0076357

**Published:** 2013-10-04

**Authors:** Jie Wang, Xingjiang Xiong, Wei Liu

**Affiliations:** Department of Cardiology, Guang’anmen Hospital, China Academy of Chinese Medical Sciences, Beijing, China; University of Buenos Aires, Argentina

## Abstract

**Background:**

Yoga is thought to be effective for health conditions. The article aims to assess the current clinical evidence of yoga for Essential hypertension (EH).

**Strategy:**

MEDLINE, EMBASE, and the Cochrane Central Register of Controlled Trials (CENTRAL) in the Cochrane Library were searched until June, 2013. We included randomized clinical trials testing yoga against conventional therapy, yoga versus no treatment, yoga combined with conventional therapy versus conventional therapy or conventional therapy combined with breath awareness. Study selection, data extraction, quality assessment, and data analyses were conducted according to the Cochrane standards.

**Results:**

A total of 6 studies (involving 386 patients) were included. The methodological quality of the included trials was evaluated as generally low. A total of 6 RCTs met all the inclusion criteria. 4 of them compared yoga plus conventional therapy with conventional therapy. 1 RCT described yoga combined with conventional therapy versus conventional therapy combined with breath awareness. 2 RCT tested the effect of yoga versus conventional therapy alone. 1 RCT described yoga compared to no treatment. Only one trial reported adverse events without details, the safety of yoga is still uncertain.

**Conclusions:**

There is some encouraging evidence of yoga for lowering SBP and DBP. However, due to low methodological quality of these identified trials, a definite conclusion about the efficacy and safety of yoga on EH cannot be drawn from this review. Therefore, further thorough investigation, large-scale, proper study designed, randomized trials of yoga for hypertension will be required to justify the effects reported here.

## Introduction

Hypertension is an important public health issue worldwide because of its high prevalence and concomitant increase in risk of disease [1,2]. Essential hypertension (EH) is a predisposing risk factor for stroke, myocardial infarction, congestive heart failure, arterial aneurysm, and the leading cause of chronic renal failure [3,4]. Approximately 90% to 95% of hypertension, affecting>1 billion adults worldwide, is the essential hypertension subtype [5]. Secondary hypertension, about 5 to 10% in hypertension, is relative to the case of primary hypertension, is refers to the secondary to renal, endocrine and nervous system disease. When found the cause and effective to remove or control the causes, secondary symptoms of high blood pressure can be cure or alleviate obviously. The prevention and management of hypertension is major public health challenges. In recent decades, different classes of antihypertensive agents were developed and tested in a variety of settings and among different patients. The studies independently and collectively contributed to a universal finding: lowering arterial pressure can remarkably reduce cardiovascular morbidity and mortality rates as well as slow the progression of renal disease, retinopathy, and all-cause deaths [6]. However, the long-term use of western medicine will produce some side effects, even produce resistance and affect therapeutic efficacy [7]. Therefore, seeking for a new effective decompression method is an important subject of hypertension treatment.

Complementary and alternative medicine (CAM) is becoming increasingly popular [8-15] and numerous interventions are regularly recommended for lowering elevated blood pressure (BP) [16-20]. Yoga, as a complementary therapeutic regimen, has been shown to be useful to individuals with a wide range of health conditions, including cardiovascular diseases (CVDs) and diabetes [21-23]. Yoga represents a body of practices with an ancient history originally derived from India, and is gaining increasing popularity in many countries around the world, consisting of various postures (Asana), breathing and meditation techniques (Pranayama). After thousands of years of evolution, yoga has spawned many factions. The orthodox India "classical yoga" includes Chi yoga, karma yoga, hatha yoga, Raja Yoga and Kundalini Yoga. There is a big difference between the different yoga theories. Chi yoga to promote a culture of knowledge; karma yoga advocate the inner practice, leading more perfect behavior; Hatha Yoga includes the spirit system and the body system; Raja yoga is mind and breath. Recently, healthcare providers have gradually become aware of yoga for its ability to improve health conditions, and numerous interventions have been developed that take full advantage of its benefits. However, yoga has not been widely recognized for hypertension. Early studies on yoga intervention for hypertension investigated the value of total body relaxation postures, primarily Savasana [24,25]. However, small numbers of subjects were utilized in the studies and there were no intervention control groups. The following, more recent studies were better controlled and conducted with sufficient numbers of subjects. For seeking the best current clinical evidence of yoga in making decisions for hypertensive patients, an increasing number of systematic reviews (SRs) and meta-analysis have been conducted to assess the efficiency of yoga for hypertension [26-28]. It is found out that yoga could contribute to low BP smoothly, reduce risk factors of hypertension (overweight, high glucose level and high cholesterol), and improve symptoms especially [29-31].

Nowadays, Yoga used alone or combined with conventional therapy has been widely used as an alternative and effective method for the treatment of EH all over the world. There is a need to provide a better recognition of yoga by the health care community as a complement to conventional medical care. And until now a number of clinical studies of yoga reported the clinical effect ranging from case reports and case series to controlled observational studies and randomized clinical trials. However, there is no critically appraised evidence such as systematic reviews or meta-analyses on potential benefits and harms of yoga for essential hypertension to justify their clinical use and their recommendation. The article aims to assess the current clinical evidence of yoga for essential hypertension.

## Methods

The supporting PRISMA checklist is available as supporting information; See Checklist S1.

### 1: Database and Search Strategies

Literature searches were conducted in MEDLINE (via Ovid, 1960 to week 26 2013, 28/June/13), EMBASE (via Dialog 1990 to 2013 week 26, 30/June/13), and the Cochrane Central Register of Controlled Trials (CENTRAL) in the Cochrane Library (June, 2013). We also searched the reference list of retrieved papers. All of those searches ended on 30 June, 2013. Ongoing registered clinical trials were searched in the website of international clinical trial registry by U.S. national institutes of health (http://clinicaltrials.gov/). The following search terms were used individually or combined: ‘hypertension’, ‘essential hypertension’, ‘Yoga’, ‘clinical trial’, and ‘randomized controlled trial’. The bibliographies of included studies were searched for additional references. The details for the full search strategy were listed in a flow diagram, as shown in Figure S1.

### 2: Inclusion Criteria

All the parallel randomized controlled trials (RCTs) of all the prescriptions based on “Yoga” compared with conventional therapy or no treatment in patients with hypertension were included. RCTs combined “Yoga” with conventional therapy compared with conventional therapy were included as well. In addition, RCTs based on yoga in combination with conventional therapy compared with conventional therapy combined with breath awareness in patients with essential hypertension were included. There were no restrictions on population characteristics, language, the type of yoga and publication type. The main outcome measure was blood pressure. Duplicated publications reporting the same groups of participants were excluded.

### 3: Data Extraction and Quality Assessment

Two authors conducted the literature searching (W. Liu, X. J. Xiong), study selection (W. Liu, X. J. Xiong), and data extraction (X. J. Xiong, W. Liu) independently. The extracted data included authors, year of publication, study size, age and sex of the participants, treatment process, details of the control interventions, outcomes, and adverse effects for each study (Table S1). Disagreement was resolved by discussion and reached consensus through a third party (J. Wang).

The methodological quality of trials was assessed independently using criteria from the Cochrane Handbook for Systematic Review of Interventions, Version 5.1.0 (W. Liu, X. J. Xiong) [32]. The items included random sequence generation (selection bias), allocation concealment (selection bias), blinding of participants and personnel (performance bias), blinding of outcome assessment (detection bias), incomplete outcome data (attrition bias), selective reporting (reporting bias), and other bias. The quality of all the included trials was categorized to low/unclear/high risk of bias (“Yes” for a low of bias, “No” for a high risk of bias, “Unclear” otherwise). Then trials were categorized into three levels: low risk of bias (all the items were in low risk of bias), high risk of bias (at least one item was in high risk of bias), unclear risk of bias (at least one item was in unclear).

### 4: Data Synthesis

Revman 5.1 software provided by the Cochrane Collaboration was used for data analyses. Continuous outcome will be presented as mean difference (MD) and its 95% CI. Statistical heterogeneity was evaluated with the Cochran chi-square (χ^2^ or Chi^2^) and quantified with *I* ^2^. Statistical heterogeneity was assessed according to the 'Cochrane Handbook of Systematic Review of Interventions' (Version 5.1.0, chapter 9.5.2). A rough guide to interpretation is as follows: 0% to 40%: might not be important; 30% to 60%: may represent moderate heterogeneity; 50% to 90%: may represent substantial heterogeneity; 75% to 100%: considerable heterogeneity. Fixed effects model was used if there is no significant heterogeneity of the data; random effects model was used if significant heterogeneity existed. In order to deal with heterogeneity and perform secondary analysis, subgroup analysis was necessary. Publication bias would explored by funnel plot asymmetry, if sufficient studies were found.

## Results

### 1: Description of Included Trials

A flow chart depicted the search process and study selection (Flow Diagram S1). After primary searches from the databases, 141 citations in MEDLINE, 17 citations in EMBASE, and 3 citations in the Cochrane Central Register of Controlled Trials (CENTRAL) in the Cochrane Library were screened. After reading the titles and abstracts, a majority of them was excluded. Full texts of 26 articles were retrieved, and 20 articles were excluded with reasons listed as shown in Table S2: participants did not meet the inclusive criteria (n = 8), duplication (n = 2), no control group (n = 3), and no data for extraction (n = 7). In the end, 6 RCTs [33-38] were included. All the RCTs were published in English.

The bibliographic details of these trials are given in Table S1. A total of 386 participants with essential hypertension were included, with the average number of 64 per trial, ranging from 30 to 90. There was a wide variation in the age of subjects (20-75 years). Only two of the six trials [34,37] specified the diagnostic criteria of hypertension of Seventh Report of the Joint National Committee on Prevention, Detection, Evaluation, and Treatment of High Blood Pressure (JNC 7).

Interventions included pranayama, asana and meditation yoga (Iyengar yoga, vajrasana, sukhasana, shavasana, “om” meditation, et al.) alone, or combined with conventional therapy. Four RCTs [33,35–37] used pranayama plus asana, one [34] employed pranayama, asana and meditation yoga and one [38] made use of pranayama alone. The controls included internal medicine care, controlling diet, regulating work and rest, changing lifestyle, an enhanced usual care (EUC), breath awareness and no treatment, etc. The total treatment duration ranged from 6 to 12 weeks. All of the 6 trials used the BP as the outcome measure.

### 2: Methodological Quality of Included Trials

The methodological quality of most included trials was generally “poor”. The details are as shown in Table S3. The randomized allocation of participants was mentioned in all trials. However, none of trials stated the method for sequence generation including random number table and allocation concealment. However, insufficient information was provided to judge whether or not it was conducted properly. Blinding of participants and personnel and blinding of outcome assessment were not mentioned in all trials. However, in yoga trials, practitioner blinding is impossible. 4 trials reported drop-out or withdraw [33,35-37] and 1 trial reported the reasons [33]. None of trials had a pre-trial estimation of sample size. 1 trial reported information on follow-up [35]. Selective reporting was generally unclear in the RCTs due to the inaccessibility to the trial protocol.

### 3: Details of included trials

6 RCTs were included in the group of studies of patients with essential hypertension ( [33-38]). However, we found that there would be a risk of bias because of studies with low methodological quality [32]. The effect estimates of yoga were shown in the Figures S1-S2.

#### 3.1: Yoga combined with conventional therapy versus conventional therapy

A total of 4 trials [33,34,37,38] reported the effect of yoga plus conventional therapy versus conventional therapy. 4 independent trials did show better effect: Ruth McCaffrey, et al. [33] conducted an RCT to assess the effectiveness of yoga on hypertensive persons with mild to moderate hypertension in Thailand. At the end of the trial, the experimental group significantly decreased blood pressure compared with the control group (systolic blood pressure [SBP]: mean difference -26.74mmHg, 95% confidence intervals -28.55 to -24.93; diastolic blood pressure [DBP]: -19.80, -21.05 to -18.55); Deepa T, et al. [34] tested the effect of yoga and meditation on mild to moderate essential hypertensives. The study showed a significant fall of mean blood pressure after 3 months of yoga (*P*<0.00001) (SBP: -9.92, -13.88 to -5.96; DBP: -9.83, -10.30 to -9.35); K Dhameja, et al. [37] evaluated the therapeutic effect of yoga in patients with hypertension. The yoga group showed a statistically significant but modest reduction in systolic blood pressure (3.5%) in comparison with the control group, which showed a reduction of 1.3%. A reduction of 6.5% was seen in diastolic blood pressure in subjects practicing yoga, as against a 2.7% decrease in the control group, with a confidence level of 93.9% (SBP: -3.19, -3.92 to -2.46; DBP: -3.47, -5.06 to -1.88); *Telles* S,et al. [38] discovered yoga combined western medicine showed better effect compared to western medicine groups alone (SBP: -4.37, -6.42 to -2.32; DBP: -1.47, -1.94 to -1.00).

#### 3.2: Yoga combined with conventional therapy versus conventional combined with plus breath awareness

Only one trial reported the effect of yoga in combination with conventional therapy compared with conventional therapy combined with breath awareness in patients with essential hypertension. Tellies S, et al. [38] discovered yoga only marginally reduced both SBP and DBP (SBP: -2.20, -3.81 to -0.59; DBP: -0.74, -1.44 to -0.04).

#### 3.3: Yoga versus conventional therapy

A total of two trials [35,36] reported the effect of yoga individually compared with conventional therapy. One trial [35] discovered the better effect on both SBP and DBP (SBP: -2.00, -2.45 to -1.55; DBP: -3.00, -3.45 to -2.55). The other trial [36] reported that yoga is superior to conventional therapy to reduce SBP and DBP (SBP: -9.60, -10.46 to -8.74; DBP: -16.36, -17.65 to -15.07).

#### 3.4: Yoga versus no treatment

Only one trial [36] showed yoga individually versus no intervention. There were statistically significant differences on the yoga group to no intervention using alone (SBP: -29.17,-29.92 to -28.42; DBP: -24.28, -24.89 to -23.67).

### 4: Adverse Effect

The safety problem about medical measurement is getting increasing concern all over the world. In our review, only one trial [35] described the adverse events even without detailed information in the treatment group, and one trial [38] reported that none of the participants have any adverse events during yoga practice. 4 trials [33,34,36,37] did not mention whether they had monitored adverse effects or not.

### 5: Publication Bias

A simple analysis of funnel plots provides a useful test for the likely presence of bias in meta-analyses. However, as the capacity to detect bias will be limited when meta-analyses are based on a limited number of small trials, the results from such analyses should be treated with considerable caution [39]. The number of included trials (6 trials) was too small to conduct any sufficient additional analysis of publication bias.

## Discussion

Due to the potential side effects of antihypertensive drugs, CAM has been favored by people all over the world. Currently, with increasing popularity of CAM, more and more systematic reviews (SRs) and meta-analysis have been conducted to assess the efficiency of CAM for EH [40]. As an important composition of CAM, yoga has made great contributions to the health and well-being of the people for their unique advantages in preventing and curing diseases, rehabilitation and health care.

Although there are numerous published studies investigating the effects of various forms of yoga on hypertension, most of these were not RCTs. There are only five randomized controlled trials of any form of yoga for EH [33-38]. The studies in this review demonstrated that yoga may have beneficial effects for a variety of populations on hypertension integrating the body and mind or alone. The result showed yoga as an adjunctive treatment to conventional therapy or no treatment significantly could lower SBP and DBP in patients with hypertension. However, the definite conclusion cannot be drawn from this paper because of the following limitations.

Firstly, all the included trials were of poor methodology quality, which were in accordance with previous studies [21,22,30]. Five trials included in this paper had risk of bias in terms of design, reporting, and methodology. They provided only inadequate reporting of study design, allocation sequence, allocation concealment, blinding, intention to treat analysis and drop outs account in the majority of trials. Randomization was mentioned, however, none of RCTs stated randomization procedure, and provided insufficient information to judge whether randomization was conducted properly, they just mentioned that ‘the patients were randomized into two or three groups’ with no further information. In addition, all the trials did not describe the blinding in details. We understood that it was difficult to perform double-blinding because of certain features associated with yoga, but blinding to the outcome assessors and data analyzer could be feasible. Unfortunately, none of the RCTs mentioned blinding to the outcome assessors or data analyzer. It directly led to performance bias and detection bias due to patients and researchers being aware of the therapeutic interventions for the subjective outcome measures. Allocation concealment was not mentioned. Therefore, we could not exclude the possibility that some of these claimed RCTs are not real RCTs. Not only that, all trials specified the diagnostic criteria of hypertension without two [34,37], four RCTs included the patients with Stage 1 hypertension [34,35,37,38], and others were Stage 2 hypertension [33,36]. So selective reporting bias might exist in this conclusion, which will reduce the homogeneity of the research objects. All the RCTs prohibited us to perform meaningful sensitivity analysis. All the included trials were not multicenter, large scale RCTs.

Secondly, the review found inadequate reporting on adverse events in the included trials. Only one trial reported adverse event [35], but without detailed information. Therefore, a conclusion about the safety of yoga cannot be made clearly. It is widely believed that it is safe to use yoga for a variety of diseases all over the world. However, parts of the practitioners would have appeared vertebral cartilage (disc) degradation, wear, etc, if they have practiced yoga for long-term. However, the short-term adverse events, such as hyperventilation used by some yoga techniques, could be occurred. In order to proper assess the safety of yoga, large-scale clinical trials with long-term follow-up are required.

Thirdly, we were unable to explore the publication bias using the funnel plot because each comparison had insufficient studies (less than 10), but the publication bias might be existed in this paper despite the positive [33-38].

Last but not least, there is a lack of information about types of control and duration of yoga, which is a quite common problem in clinical trials. Future trials should provide information about standardization including types of control, detailed regimen, and duration of treatment.

In summary, there is some encouraging evidence of yoga for lowering SBP and DBP. However, due to unclear methodological quality of these identified trials, a definite conclusion about the efficacy and safety of yoga on EH cannot be drawn from this review. Therefore, further thorough investigation, large-scale, proper study designed, randomized trials of yoga for hypertension will be required to justify the effects reported here. To ensure evidence-based clinical practice, further rigorous placebo-controlled, randomized trials are warranted. Specially, they should assure adequate concealment and sequence of allocation, blinding of outcome assessors and use functional outcome as the primary outcome measured at long-term follow-up. Furthermore, descriptions of withdrawal/dropout during the trial and use of intention-to-treat analysis play an important role, reports of the trials should conform to the recommendations of the CONSORT statement [41]. If reliable RCT results confirmed yoga positive effects for treatment of hypertension, it would be blessing news to use complementary and alternative medicine for hypertension.

## Supporting Information

Checklist S1 **PRISMA 2009 checklist.**
(DOC)

Flow Diagram S1 **PRISMA 2009 Flow Diagram.**
(DOCX)

Figure S1 **The forest plot of outcome measure SBP.**
(EPS)

Figure S2 **The forest plot of outcome measure DBP.**
(EPS)

Table S1 **Characteristics and methodological quality of included studies.**
(DOCX)

Table S2 **Excluded studies with reasons.**
(DOC)

Table S3 **Quality assessment of included randomized controlled trials.**
(DOC)
