# Supplementary material for: Yoga for Essential Hypertension: A Systematic Review
Source: PLoS One. 2013 Oct 4;8(10):e76357. doi: 10.1371/journal.pone.0076357 (PMC3790704; doi:10.1371/journal.pone.0076357)
Supplement: Flow Diagram S1 — PRISMA 2009 Flow Diagram. (DOCX) [file pone.0076357.s002.docx]

**PRISMA 2009 Flow Diagram**

Full-text articles assessed for eligibility

(n = 26)

20 articles were excluded with reasons listed as the following.

Participants did not meet the inclusive criteria (n = 8)

Duplication (n = 2)

No control group (n =3)

No data for extraction (n =7)

**Identification**

**Screening**

**Eligibility**

**Included**

Records identified through database searching

(n =161)

Additional records identified through other sources

(n = 10)

Records after duplicates removed

(n =122)

Records screened

(n =122)

Records excluded

(n =96)

PRISMA 2009 Flow Diagram

Studies included in the review (n = 6)

Studies included in quantitative synthesis (meta-analysis)
(n = 6)
