# Supplementary material for: Yoga for Essential Hypertension: A Systematic Review
Source: PLoS One. 2013 Oct 4;8(10):e76357. doi: 10.1371/journal.pone.0076357 (PMC3790704; doi:10.1371/journal.pone.0076357)
Supplement: Table S1 — Characteristics and methodological quality of included studies. (DOCX) [file pone.0076357.s005.docx]

**Table S1: Characteristics and methodological quality of included studies.**

| Study ID | | Sample(F/M) | Age(yrs) | Diagnosis  standard | Intervention | Control | Course  (week) | Outcome measure |
| --- | --- | --- | --- | --- | --- | --- | --- | --- |
| Ruth McCaffrey, et al.  2005 [33] | | 61  T:17/10; C:18/9 | T:56.7; C:56.2 | Hypertension diagnostic criteria (unclear) | Yoga(asana, pranayama)  +routine outpatient care | routine outpatient care | 8  (3 times/week) | BP |
| Deepa T  et al.  2012 [34] | | 30  T:7/8; C:7/8 | 45 to 65 (T/C not reported) | Seventh Report of the Joint National Committee on Prevention, Detection,  Evaluation, and Treatment of High Blood Pressure ( JNC 7) | Yoga(asana, pranayama, meditation)+an-tihypertensive drugs | anti-hypertensive drugs | 12  (5 days/week；45 minutes per day) | BP |
| Debbie L. Cohen , et al.  2011 [35] | | 78  T:23/23; C:16/16 | T:48.2±1.6  C:48.3±2.4 | Hypertension diagnostic criteria (unclear) | Iyengar Yoga(asana, pranayama) | an enhanced usual care (EUC) | 12  (twice weekly 70 min for the first 6 weeks and once a week for the following 6 weeks) | BP;  Adverse even |
| R.Murugesan, 2000 [36] | 33  T:11  C1:11  C2:11(F/M not reported) | | 35 to 65 (T/C not reported) | Hypertension diagnostic criteria (unclear) | Yoga | C1:no treatment  C2:anti-hypertensive drugs | 11 (morning and evening 1hr each day 6 days a week.) | BP |
| Kanupriya Dhameja, et al.  2012[37] | 60  T:30  C:30  (F/M not reported) | | T:48.07±8.30  C:50.33±158.40 | Seventh Report of the Joint National Committee on Prevention, Detection,  Evaluation, and Treatment of High Blood Pressure ( JNC 7) | Yoga(asana, pranayama) + conventional therapy | conventional therapy | 6 (50–60 minutes per day) | BP |
| [Telles S](http://www.ncbi.nlm.nih.gov/pubmed?term=Telles%20S%5BAuthor%5D&cauthor=true&cauthor_uid=23334063),et al.  2013[38] | 90 T:30  C1:30  C2:30(F/M: 30/60) | | 49.7±9.5 (T/C not reported) | Hypertension diagnostic criteria (unclear) | Yoga(pranayama)+ conventional therapy | C1:conventional therapy + breath awareness  C2:conventional therapy | 8 (10 minutes per day) | BP; Adverse even |

Abbreviations: T, Intervention group; C, control group
