# Supplementary material for: Yoga for Essential Hypertension: A Systematic Review
Source: PLoS One. 2013 Oct 4;8(10):e76357. doi: 10.1371/journal.pone.0076357 (PMC3790704; doi:10.1371/journal.pone.0076357)
Supplement: Table S2 — Excluded studies with reasons. (DOC) [file pone.0076357.s006.doc]

## Table S2. Excluded studies with reasons

| **Study** | **Reason for exclusion** |
| --- | --- |
| [Bijlani RL](javascript:void(null))2005^1^ | Participants did not meet the inclusive criteria |
| [Narendran S](javascript:void(null)) 2005^2^  VaishaliV.Agte2011^3^ | Participants did not meet the inclusive criteria  Participants did not meet the inclusive criteria |
| [Dolgoff-Kaspar R](http://www.ncbi.nlm.nih.gov/pubmed?term=Dolgoff-Kaspar R%5BAuthor%5D&cauthor=true&cauthor_uid=22894892) 2012^4^  Vaishali V. Agte 2011^5^  [Cohen DL](http://www.ncbi.nlm.nih.gov/pubmed?term=Cohen DL%5BAuthor%5D&cauthor=true&cauthor_uid=23721984) 2013^6^  Jayashree R 2013^7^  Miles SC 2013^8^ | Participants did not meet the inclusive criteria  Participants did not meet the inclusive criteria  Participants did not meet the inclusive criteria  Participants did not meet the inclusive criteria  Participants did not meet the inclusive criteria |
| [Rakhshani A](javascript:void(null)) 2012^9^ | Duplication |
| [Khalsa SB](javascript:void(null))  2004^10^ | Duplication |
| [Vijayalakshmi P](javascript:void(null)) 2004^11^ | No control group |
| [Santaella DF](javascript:void(null)) 2006^12^ | No control group |
| S.N. Murthy 2011^13^ | No control group |
| [Lee SW](javascript:void(null)) 2004^14^ | No data for extraction |
| [Sharma R](javascript:void(null)) 2008^15^ | No data for extraction |
| Mourya M 2009^16^  Blom K 2012^17^  Sheng-Chia Chung 2011^18^  [Julio Mizuno](http://www.bodyworkmovementtherapies.com/article/S1360-8592(12)00228-8/abstract) 2013 ^19^  Ziv A 2013^20^ | No data for extraction  No data for extraction  No data for extraction  No data for extraction  No data for extraction |

1. [Bijlani RL](javascript:void(null))；[Vempati RP](javascript:void(null))；[Yadav RK](javascript:void(null)), et al.(2005)A brief but comprehensive lifestyle education program based on yoga reduces risk factors for cardiovascular disease and diabetes mellitus. [J Altern Complement Med 11](javascript:void(null)) :267-74.
2. [Narendran S](javascript:void(null))；[Nagarathna R](javascript:void(null))；[Gunasheela S](javascript:void(null))；[Nagendra HR](javascript:void(null)) (2005 Efficacy of yoga in pregnant women with abnormal Doppler study of umbilical and uterine arteries . [J Indian Med Assoc103](javascript:void(null)) :12-4, 16-7.
3. Vaishali V. Agte, Madhavi U, Jahagirdar, et al. (2011) The effects of sudarshan kriya yoga on some physiological and biochemical parameters in mild hypertensive patients. Indian J Physiol Pharmacol 55:183-7.
4. [Dolgoff-Kaspar R](http://www.ncbi.nlm.nih.gov/pubmed?term=Dolgoff-Kaspar R%5BAuthor%5D&cauthor=true&cauthor_uid=22894892), [Baldwin A](http://www.ncbi.nlm.nih.gov/pubmed?term=Baldwin A%5BAuthor%5D&cauthor=true&cauthor_uid=22894892), [Johnson MS](http://www.ncbi.nlm.nih.gov/pubmed?term=Johnson MS%5BAuthor%5D&cauthor=true&cauthor_uid=22894892), et al (2012) Effect of laughter yoga on mood and heart rate variability in patients awaiting organ transplantation: a pilot study. [Altern Ther Health Med](http://www.ncbi.nlm.nih.gov/pubmed/22894892) 18(5): 61-6.
5. Vaishali V. Agte, Madhavi U, Jahagirdar, et al. (2011) The effects of sudarshan kriya yoga on some physiological and biochemical parameters in mild hypertensive patients, Indian J Physiol Pharmacol 55:183-7.
6. [Cohen DL](http://www.ncbi.nlm.nih.gov/pubmed?term=Cohen DL%5BAuthor%5D&cauthor=true&cauthor_uid=23721984), [Bowler A](http://www.ncbi.nlm.nih.gov/pubmed?term=Bowler A%5BAuthor%5D&cauthor=true&cauthor_uid=23721984), [Fisher SA](http://www.ncbi.nlm.nih.gov/pubmed?term=Fisher SA%5BAuthor%5D&cauthor=true&cauthor_uid=23721984), et al. (2013) [Lifestyle Modification in Blood Pressure Study II (LIMBS): Study protocol of a randomized controlled trial assessing the efficacy of a 24week structured yoga program versus lifestyle modification on blood pressure reduction,](http://www.ncbi.nlm.nih.gov/pubmed/23721984) [Contemp Clin Trials](http://www.ncbi.nlm.nih.gov/pubmed/?term=Lifestyle+Modification+in+Blood+Pressure+Study+II+(LIMBS)%3A+Study+protocol+of+a+randomized+controlled+trial+assessing+the+efficacy+of+a+24week+structured+yogaprogram+versus+lifestyle+modification+on+blood+pressure+reduction.) 36 (1): 32-40.
7. Jayashree R, Malini A, Rakhshani A, et al. (2013) [Effect of the integrated approach of yoga therapy on platelet count and uric acid in pregnancy: A multicenter stratified randomized single-blind study,](http://www.ncbi.nlm.nih.gov/pubmed/23440456) [Int J Yoga](http://www.ncbi.nlm.nih.gov/pubmed/?term=Effect+of+the+integrated+approach+of+yoga+therapy+on+platelet+count+and+uric+acid+in+pregnancy%3A+A+multicenter+stratified+randomized+single-blind+study.) 6 (1):39-46.
8. Miles SC, Chun-Chung C, Hsin-Fu L, et al. (2013) [Arterial blood pressure and cardiovascular responses to yoga practice,](http://www.ncbi.nlm.nih.gov/pubmed/23341425) Altern Ther Health Med 19 (1):38-45.
9. [Rakhshani A](javascript:void(null))；[Nagarathna R](javascript:void(null))；[Mhaskar R](javascript:void(null))；[Mhaskar A](javascript:void(null)), et al. (2012) The effects of yoga in prevention of pregnancy complications in high-risk pregnancies: A randomized controlled trial. [Prev Med 55](javascript:void(null)):333-40.
10. [Khalsa SB](javascript:void(null))  (2004) Yoga as a therapeutic intervention: a bibliometric analysis of published research studies. [Indian J Physiol Pharmacol 48](javascript:void(null)) :269-85.
11. [Vijayalakshmi P](javascript:void(null))；[Madanmohan](javascript:void(null))；[Bhavanani AB](javascript:void(null)) (2004) Modulation of stress induced by isometric handgrip test in hypertensive patients following yogic relaxation training. [Indian J Physiol Pharmacol. 48](javascript:void(null)):59-64
12. [Santaella DF](javascript:void(null))；[Araujo EA](javascript:void(null))；[Ortega KC](javascript:void(null)) (2006) Aftereffects of exercise and relaxation on blood pressure. [Clin J Sport Med 16](javascript:void(null)) :341-7
13. S.N. Murthy, N.S.N. Rao, Babina Nandkumar, et al. (2011) Role of naturopathy and yoga treatment in the management of hypertension. Complementary Therapies in Clinical Practice 17：9-12.
14. [Lee SW](javascript:void(null))；[Mancuso CA](javascript:void(null))；[Charlson ME](javascript:void(null))  (2004) Prospective study of new participants in a community-based mind-body training program. Indian J Physiol Pharmacol. [J Gen Intern Med 19](javascript:void(null)) :760-5.
15. [Sharma R](javascript:void(null))；[Gupta N](javascript:void(null))；[Bijlani RL](javascript:void(null)) (2008) Effect of yoga based lifestyle intervention on subjective well-being. J Altern Complement Med 15:711-7.
16. Monika Mourya, Aarti Sood Mahajan, Narinder Pal Singh (2009) Effect of Slow- and Fast-Breathing Exercises on Autonomic Functions in Patients with Essential Hypertension. [J Altern Complement Med 15](javascript:void(null)): 711-7.
17. Blom K, How M, Dai M, et al. (2012) Hypertension Analysis of stress reduction using Mindfulness meditation and Yoga (The HARMONY Study): study protocol of a randomised control trial.BNJ Open 2: e000848.
18. Sheng-Chia Chung, Maria M. Brooks, Madhur Rai, et al. (2012) Effect of Sahaja Yoga Meditation on Quality of Life, Anxiety, and Blood Pressure Control. [J Altern Complement Med](javascript:void(null)) 18: 589-96.
19. [Julio Mizuno](http://www.bodyworkmovementtherapies.com/article/S1360-8592(12)00228-8/abstract), [Henrique Luiz Monteiro](http://www.bodyworkmovementtherapies.com/article/S1360-8592(12)00228-8/abstract). (2013) An assessment of a sequence of yoga exercises to patients with arterial hypertension, [J Bodyw Mov Ther](http://www.ncbi.nlm.nih.gov/pubmed/?term=An+assessment+of+a+sequence+of+yoga+exercises+to+patients+with+arterial+hypertension) 17 (1):35-41.
20. Ziv A, Vogel O, Keret D, et al. (2013) [Comprehensive Approach to Lower Blood Pressure (CALM-BP): a randomized controlled trial of a multifactorial lifestyle intervention,](http://www.ncbi.nlm.nih.gov/pubmed/23595161) J Hum Hypertens 2013 Apr 18. doi: 10.1038/jhh.
