# Supplementary material for: Yoga for Essential Hypertension: A Systematic Review
Source: PLoS One. 2013 Oct 4;8(10):e76357. doi: 10.1371/journal.pone.0076357 (PMC3790704; doi:10.1371/journal.pone.0076357)
Supplement: Table S3 — Quality assessment of included randomized controlled trials. (DOC) [file pone.0076357.s007.doc]

**Table S3**: Quality assessment of included randomized controlled trials.

| Included trials | | Random sequence generation | Allocation concealment | Blinding of participants and personnel | | Blinding of outcome assessment | Incomplete outcome data | Selective reporting | Other sources of bias | Risk of bias |
| --- | --- | --- | --- | --- | --- | --- | --- | --- | --- | --- |
| Ruth McCaffrey  2005 [33] | Unclear | | Unclear | | Unclear | Unclear | Yes | No | Unclear | High |
| Deepa T  et al.2012 [34] | Unclear | | Unclear | | Unclear | Unclear | No | No | Unclear | High |
| Debbie L. Cohen  2011 [35] | | Unclear | Unclear | | Unclear | Unclear | Yes | No | Unclear | High |
| R.Murugesan, 2000 [36] | | Unclear | Unclear | | Unclear | Unclear | Yes | No | Unclear | High |
| Kanupriya Dhameja  2012[37] | | Unclear | Unclear | | Unclear | Unclear | Yes | No | Unclear | High |
| [Telles S](http://www.ncbi.nlm.nih.gov/pubmed?term=Telles S%5BAuthor%5D&cauthor=true&cauthor_uid=23334063),et al.  2013[38] | | Unclear | Unclear | | Unclear | Unclear | Yes | No | Unclear | High |
